# Blood pressure and outcome after aneurysmal subarachnoid hemorrhage

**DOI:** 10.1038/s41598-022-11903-4

**Published:** 2022-05-14

**Authors:** Marvin Darkwah Oppong, Lisa Steinwasser, Christoph Rieß, Karsten H. Wrede, Thiemo F. Dinger, Yahya Ahmadipour, Philipp Dammann, Laurèl Rauschenbach, Meltem Gümüs, Cornelius Deuschl, Ulrich Sure, Ramazan Jabbarli

**Affiliations:** 1 Department of Neurosurgery and Spine Surgery, University Hospital Essen, University of Duisburg-Essen, 45147 Essen, Germany; 2 Department of Diagnostic and Interventional Radiology and Neuroradiology, University Hospital Essen, University of Duisburg-Essen, Essen, Germany

**Keywords:** Cerebrovascular disorders, Stroke

## Abstract

Blood pressure management is crucial in the treatment of patients with aneurysmal subarachnoid hemorrhage (aSAH). Possible association between the blood pressure increase and the risk of delayed cerebral ischemia (DCI) and different systemic complications after aSAH is still a matter of debate. This study aims to elucidate the influence of blood pressure levels on the outcome of aSAH. All consecutive aSAH patients (n = 690) treated between 01/2003 and 06/2016 were included. The mean value of the mean arterial pressure (MAP) during 14 days after ictus was calculated for each individual. According to the institutional standards of vasospasm management, the mean 14 days MAP ≥ 95 mmHg was referred as increased (IMAP) and the patients with and without vasospasm were analyzed separately. Study endpoints were the occurrence of DCI on computed tomography scans, development of cardiac and nephrological complications, and poor outcome 6 months after aSAH (mRS > 2). Associations were tested in univariable/multivariable binary logistic regression analysis. IMAP was documented in 474 (68.7%) cases and was more common in individuals with poor neurological conditions at admission (p < 0.001), severe amount of intracranial blood (p = 0.001) and premorbid hypertension (p < 0.001). IMAP was independently associated with the occurrence of DCI (p = 0.014; aOR = 2.97; 95% CI 1.25–7.09) and poor functional outcome (p = 0.020; aOR = 3.14; 95% CI 1.20–8.22) in patients with vasospasm, but not in counterparts without vasospasm (p = 0.113/p = 0.086). IMAP had no influence on cardiac or nephrological complications. In aSAH individuals with cerebral vasospasm, sustained increase of blood pressure exceeding the therapeutic targets is strongly associated with the risk of DCI and poor outcome. Therefore, such an intrinsic increase of blood pressure might reflect the autoregulatory mechanisms against the impending cerebral ischemia in patients with cerebral vasospasm.

Trial registration number: German clinical trial registry (DRKS, Unique identifier: DRKS00008749, 06/09/2015).

## Introduction

Aneurysmal subarachnoid hemorrhage (aSAH) is a subform of hemorrhagic stroke with high morbidity and mortality. Besides the initial bleeding and possible rebleeding event, patients with aSAH are endangered by multiple subsequent complications, especially during the first weeks after aneurysm rupture. One major contributor to poor outcome is delayed cerebral ischemia (DCI)^1^. Multiple factors including cerebral vasospasm, microthrombosis, activation of inflammatory pathways, and spreading cortical depolarization contribute to development of infarction in former unaffected brain areas^2^. Preventive measurements include administration of nimodipine and endovascular intervention in case of vasospasm^3,4^.

Induced hypertension had a long-standing role in the prevention of DCI and associated infarction^5,6^. However, the only randomized controlled trial so far showed that an extreme increase of the mean arterial pressure (MAP) does not result in better outcome, but is related to higher risk of cardiac complications^7,8^. According to the recent aSAH guidelines^4,9^, blood pressure management remains the cornerstone element of aSAH treatment including the avoidance of hypotension, and the maintenance of the MAP over certain cut-off values widely varying throughout the neurovascular centers.

Along with this vasospasm-targeted MAP-based blood pressure management, patients’ premorbid conditions, initial severity of bleeding, later complications, and different medical interventions during hospitalization may also contribute to blood pressure alterations after aSAH^10,11^. Under certain circumstances, MAP might exceed the therapeutic targets and necessitate the treatment with anti-hypertensive agents. In this context, the value of hypertensive and non-hypertensive blood pressure values for the course and outcome of aSAH remains unclear.

This study aims to elucidate the clinical impact of sustained blood pressure increase exceeding the aimed therapeutic frame on the risk of DCI associated infarction, different systemic complications and poor outcome after aSAH.

## Material and methods

All adult patients treated for aSAH between January 2003 and June 2016 at our neurovascular center were eligible for this study. The exclusion criteria were: (a) hospital admission later than 48 h after ictus; (b) hospital stay at our clinic for less than 72 h; (c) no aneurysm treatment; (d) mycotic aneurysm as a bleeding source; (e) no data on MAP values.

The approval of the institutional ethics committee (Ethik-Kommission, Medizinische Fakultät der Universität Duisburg-Essen, Registration number: 15-6331-BO) for this study was obtained and has been performed in accordance with the ethical standards laid down in the 1964 Declaration of Helsinki and its later amendments. The study was registered in the German clinical trial registry (DRKS, Unique identifier: DRKS00008749). All patients or their relatives gave written informed consent within the treatment contract before inclusion into the database.

### aSAH treatment: general aspects

All patients with a suspected aSAH received radiographic imaging (digital subtraction angiography (DSA) or computed tomography (CT) angiography of the head) for identification of the bleeding source. Commonly, aneurysm treatment was performed within 24 h after hospital admission by endovascular coiling or microsurgical clipping. Acute hydrocephalus was treated by insertion of an external ventricular drain allowing the measurement of the intracranial pressure (ICP). Raised ICP (> 20 mmHg) was treated conservatively by drainage of cerebrospinal fluid, head elevation, osmotherapy, deep sedation, and relaxation. Patients with increased ICP refractory to conservative management were referred to decompressive craniectomy. Cerebral perfusion pressure (CPP) was maintained over 60 mmHg. Posthemorrhagic hydrocephalus was treated with ventriculoperitoneal shunt placement. Follow-up CT scans of the head were performed in the first 24 h after aneurysm treatment, and if clinically indicated (i.e. failed weak up attempt, increased ICP, clinical deterioration).

### aSAH treatment: cerebral vasospasm and blood pressure management

All patients were administered nimodipine orally for 21 days after ictus. Fluid management included the maintenance of euvolemia. In addition, transcranial doppler (TCD) ultrasound was performed at least once a day for the first 14 days after ictus. Patients showing clinical signs of vasospasm in form of (a) TCD velocities higher than 120 m/s and compromised consciousness; (b) new neurological deficits or a decline in the Glasgow coma scale over 2 points not related to other reasons (like rebleeding or hydrocephalus) were scheduled for immediate DSA for the verification and invasive endovascular treatment. As first line therapy, pharmacologic angioplasty was performed using intraarterial nimodipine. Mechanical angioplasty (transluminal balloon angioplasty) was performed only as a rescue option in large proximal intracranial arteries. In case of recurrence of new deficits or increased TCD values, endovascular therapy was repeated up to twice daily until the resolution of vasospasm signs. Post angiographic TCD and/or clinical recovery was reevaluated to determine the success of treatment.

Until obliteration of the aneurysm, systolic blood pressure was kept < 150 mmHg using antihypertensive drugs, if needed. After aneurysm treatment, MAP was retained at ≥ 70 mmHg for the first 14 days after ictus in all aSAH individuals. In patients with confirmed clinical and angiographic vasospasm, the MAP was raised to ≥ 90 mmHg using norepinephrine administration via a central line, if necessary. In cases where the designated MAP target could not be reached with norepinephrine alone, the intensive care physician used additional vasopressors depending on the individual patient's situation. MAP target was kept for the duration of VS. In patients with an unvoluntary systolic blood pressure (SBP) over 220 mmHg, blood pressure was lowered up using antihypertensive medication without compromising the designated MAP. Blood pressure was monitored continuously via arterial line and was documented every 2 h in the patients’ chart.

### Data management

The variables of interest, including radiographic, clinical, laboratory and demographic characteristics of the patients, were collected from the electronic patients’ records and the institutional prospective aneurysm database. The radiographic data were separately reviewed by the senior author (RJ) blinded at this time for any clinical information.

Initial clinical severity of aSAH was classified using the World Federation of Neurological Surgeons (WFNS) scale^12^ and was dichotomized into good (WFNS 1–3) and poor grade (WFNS 4–5) for statistical analysis. The original Fisher scale was used to assess the radiological severity of bleeding^13^, with further dichotomization into high (Fisher 3–4) and low (Fisher 1–2) grades. Occurrence of new cerebral infarction(s) was judged upon the follow-up CT imaging up to 6 weeks after aSAH. New hypodensities visualizable within 48 h after early aneurysm occlusion were referred as early infarcts, whereas later infarct events were allocated as DCI associated infarction.

A mean value of MAP and SBP for the first 14 days after ictus were calculated using the daily mean MAP/SBP values collected from the ICU documentation system. For further statistical evaluation, the 14-days mean MAP values were dichotomized according to the institutional standards of blood pressure management after aneurysm treatment: within (MAP < 95 mmHg, standard MAP [SMAP]) and over (MAP ≥ 95 mmHg, increased MAP [IMAP]) the maximal therapeutic target. No patient showed the 14-days mean MAP value < 70 mmHg. The total dosage of used norepinephrine (in milligram [mg]) was extracted for each patient in the cohort.

**Figure 1:**
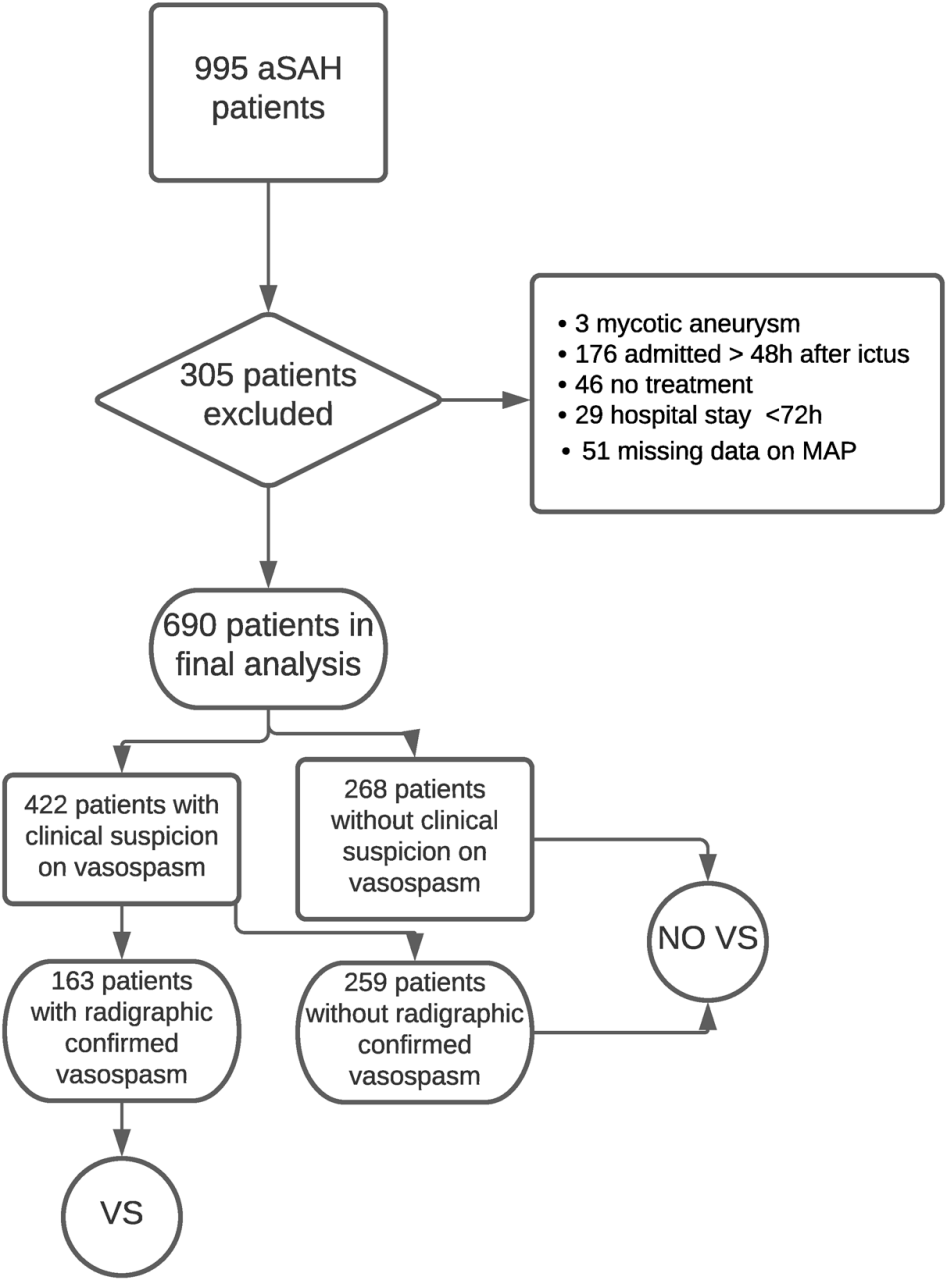
Overview of the recruitment process of the study. *aSAH *aneurysmal subarachnoid hemorrhage, *MAP *mean arterial pressure, *(NO) VS (no) vasospasm.*

Depending on the presence of clinical signs and radiographic proof of cerebral vasospasm (basal vessel constriction or perfusion deficit) the patients in the cohort were referred to vasospasm (VS) and no vasospasm (NO VS) groups (see Fig. 1 with the flow-chart). Patients in the VS group were treated with induced hypertension aiming at MAP ≥ 90 mmHg as long as the vasospasm persisted.

Along with preexisting conditions recorded from the medical charts, the occurrence of following systemic complications during the hospital treatment was also collected:

- impaired renal function defined as any decrease of the mean glomerular filtration rate under 60 ml/min/1.73 m^**2**^);
- acute coronary syndrome (ACS) based on the changes in the cardiac enzymes and/or electrocardiography (ECG); 12-channel ECG and laboratory work-up was performed if changes in continuous ECG monitoring (i.e., new atrial fibrillation) or clinical symptoms of ACS occurred.
- Finally, all chest x-rays during the first 14 days were screened to identify new pulmonary congestion not present on the admission X-ray utilizing the Shochat score (cut-off: 2 points)^14^.

The outcome was assessed at a 6-month follow-up and documented using the modified Ranking scale (mRS)^15^. An mRS > 2 was defined as poor outcome.

### Study endpoints and statistics

The correlation of the 14-days mean MAP values with the functional outcome at 6 months and DCI associated infarction development were set as the primary endpoint. Secondary endpoint included the impact of the 14-days mean MAP values on the occurrence of systemic complications during aSAH.

All statistical analyses were performed using SPSS Version 27 for Mac (IBM Corp). The significance level was set to p < 0.05. Missing data were addressed using multiple imputations.

Univariate analysis was performed to address the correlation of the 14-days mean MAP with the patients’ baseline characteristics and initial severity of aSAH as well as secondary endpoints. Chi-Square test was used for dichotomous/dichotomized variables; for samples with a size smaller than 5, the Fisher exact test was used. Continuous variables were tested with the Students t-test for normally distributed data and the Mann–Whitney-U test for non-normal distributed data. Multivariable binary logistic regression analysis was performed for the primary study endpoints to prove independent correlations. For the primary endpoints, the results were adjusted for initial clinical and radiological aSAH severity, the need for ICP treatment, presence of premorbid arterial hypertension and patients’ age. In addition, the analyses were performed for aSAH individuals in the VS and NO VS groups separately.

### Ethics approval

Ethik-Kommission, Medizinische Fakultät der Universität Duisburg-Essen, Registration number: 15-6331-BO.

## Results

A total of 690 patients were included in the final analysis (details regarding the selection process is given in Fig. 1). The majority of patients were female (67.2%), had a high radiographic severity of aSAH (90.4%), and were treated by endovascular coiling (58.7%). Three hundred ten (44.9%) patients had a poor clinical condition at initial presentation. In the whole cohort, a MAP ≥ 95 mmHg (IMAP) was observed in 474 (68.7%) individuals and occurred more commonly in patients with poor clinical condition at admission (p < 0.001; odds ratio [OR] = 2.14; 95% confidence interval [CI] 1.53–3.00), high radiographic severity (p = 0.001; OR = 2.58; 95% CI 1.53–3.00) and premorbid hypertension (p < 0.001; OR = 2.14; 95% CI 1.52–3.02, see also Table S1 in the supplementary materials for a complete overview of the cohort characteristics). Seventy-two patients died between day 3 and 14.

Clinical signs of cerebral vasospasm were suspected in 61.2%, but angiographically confirmed in 23.5% of the cohort (Fig. 1). Table 1 presents baseline characteristics of aSAH individuals with SMAP and IMAP in the VS and NO VS groups separately. The distribution of Map between the VS and NO VS group is given in Fig. S1 in the supplementary material.

**Table 1:** Univariate analysis for baseline characteristics of the patients with SMAP/IMAP in the VS and NO VS groups. Significant p-values are marked bold.

Parameter	VS group		p	OR	95% CI
	SMAP/n = 37	IMAP/n = 126			
	%/Mean ± SD	%/Mean ± SD			
**Demographics**					
Age (years)	51 ± 13	51 ± 12	0.953		
Sex (female)	75.7%	69.8%	0.491	0.74	0.32–1.73
Premorbid arterial hypertension	56.8%	73.0%	0.059	2.06	0.96–4.41
**aSAH characteristics**					
WFNS 4–5	40.5%	50.8%	0.273	1.51	0.72–3.19
Fisher 3–4	97.1%	95.2%	> 0.99	0.59	0.07–5.06
**Treatment**					
Clipping	51.4%	42.1%	0.317	0.69	0.33–1.44
ICP therapy	51.4%	56.0%	0.618	1.21	0.58–2.52
Additional vasopressors	5.4%	11.9%	0.369	2.23	0.49–10.25

	NO VS group		p	OR	95% CI
	SMAP/n = 179	IMAP/n = 348			
	%/Mean ± SD	%/Mean ± SD			
**Demographics**					
Age (years)	54 ± 15	56 ± 13	0.145		
Sex female	67.6%	65.2%	0.587	0.90	0.61–1.32
Premorbid art. hypertension	60.9%	77.6%	** < 0.001**	2.22	1.50–3.29
**aSAH characteristics**					
WFNS 4–5	30.7%	50.6%	** < 0.001**	2.31	1.58–3.38
Fisher 3–4	81.4%	92.4%	** < 0.001**	2.76	1.56–4.89
Treatment					
Clipping	41.9%	39.7%	0.619	0.91	0.63–1.32
ICP therapy	34.8%	50.6%	**0.001**	1.91	1.32–2.78
Additional vasopressors	10.7%	11.0%	0.897	1.04	0.58–1.86

*95% CI *95% confidence interval, *ACS *acute coronary syndrome, *aSAH *aneurysmal subarachnoid hemorrhage, *ICP *intracranial pressure, *(I/S)MAP *(increased/standard) mean arterial pressure, *OR *odds ratio,* SD *standard deviation, *(NO) VS *(no) vasospasm, *WFNS *World Federation of Neurosurgical Societies*.*

### MAP and functional outcome

**Figure 2:**
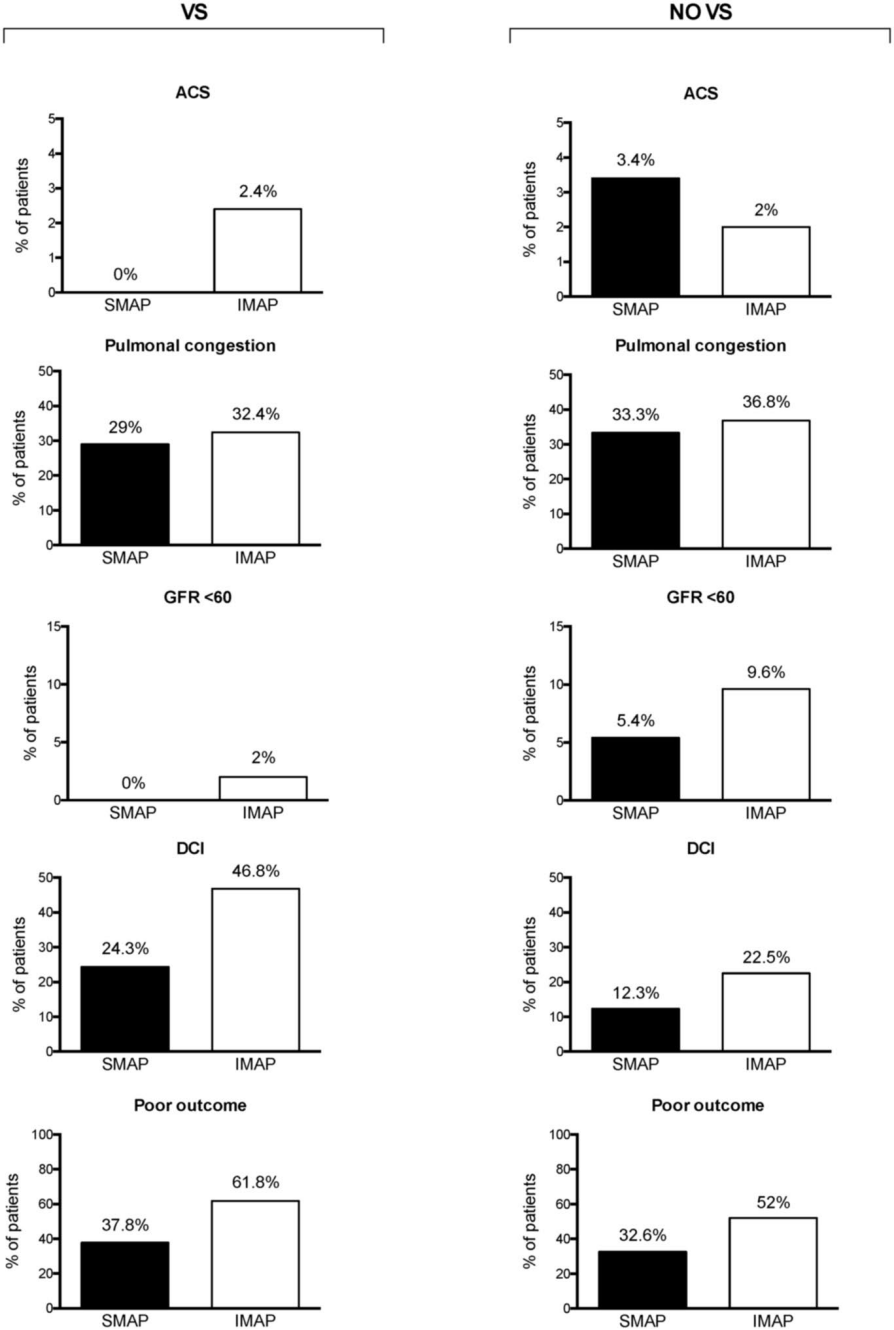
Distribution of the rates of ACS, kidney dysfunction (GFR < 60 mm/min/1.73 m^**2**^), pulmonal congestion (Shochat score > 1), DCI associated infarction and poor outcome between the individuals with SMAP and IMAP in the VS and NO VS groups. More detailed data on the associations between the 14-days MAP and the occurrence of the study endpoints are presented in the supplementary table S2. *ACS *acute coronary syndrome,* aSAH *aneurysmal subarachnoid hemorrhage, *DCI *delayed cerebral ischemia, *(I/S)MAP *(increased/standard) mean arterial pressure, *(NO) VS *(no) vasospasm.

Poor outcome was observed in 48.0% of the patients in the cohort. In multivariable analysis, IMAP (p = 0.020; adjusted [a]OR = 3.14; 95% CI 1.20–8.22) was independently associated with poor outcome in the VS group (see Table 2, Fig. 2) after correction for age, radiographic or clinical severity of aSAH, increased ICP and premorbid hypertension. In the NO VS group, no independent impact on the functional outcome could be shown for IMAP (p = 0.086, Fig. 2).

**Table 2:** Multivariate analysis for the influence of IMAP on poor outcome and DCI occurrence. Significant p-values are marked bold.

	VS group			NO VS group		
Parameter	p	aOR	95% CI	p	aOR	95% CI
**DCI associated infarction**						
Age (years)	**0.019**	1.04	1.01–1.07	**0.009**	1.02	1.01–1.04
WFNS 4/5	0.538	1.24	0.63–2.46	**0.003**	2.12	1.30–3.48
Fisher ¾	0.655	0.69	0.13–3.54	0.122	2.52	0.78–8.14
Art. Hypertension	0.461	0.75	0.36–1.60	0.400	0.80	0.47–1.35
ICP therapy	0.062	1.96	0.97–3.95	** < 0.001**	2.90	1.75–4.81
IMAP	**0.014**	2.97	1.25–7.09	0.113	1.56	0.90–2.70
**Poor outcome**						
Age (years)	** < 0.001**	1.09	1.04–1.14	** < 0.001**	1.06	1.04–1.08
WFNS 4/5	** < 0.001**	5.17	2.28–11.75	** < 0.001**	4.35	2.75–6.89
Fisher ¾	0.969	1.04	0.14–7.58	**0.031**	3.17	1.11–9.02
Art. Hypertension	0.773	1.14	0.47–2.75	0.395	1.25	0.75–2.11
ICP therapy	** < 0.001**	4.81	2.03–11.39	** < 0.001**	7.37	4.55–11.92
IMAP	**0.020**	3.14	1.20–8.22	0.086	1.53	0.94–2.47

*95% CI *95% confidence interval, *DCI *delayed cerebral ischemia, *ICP *intracranial pressure, *IMAP *increased mean arterial pressure, *aOR *adjusted odds ratio, *(NO) VS *(no) vasospasm, *WFNS *World Federation of Neurosurgical Societies.

### MAP and occurrence of DCI associated infarction

Follow-up CT scans identified DCI infarction in 24.4% of the cases. In multivariable analysis, IMAP (p = 0.014; aOR = 2.97; 95% CI 1.25–7.09) was independently associated with the occurrence of DCI associated infarction in the VS, but not in the NO VS group (p = 0.113, Table 2, Fig. 2).

### MAP and systemic complications during aSAH

The total dosage of norepinephrine used in the first 14 days was significantly higher in the NO VS group for IMAP (p = 0.007; median: 29.58 mg (interquartile range: 4.24–149.56 mg) vs. 23.2 mg (interquartile range:1.12–100.51 mg), but not in the VS group (p = 0.787, supplemental Fig. S2). The total use of additional vasopressors was not increased for patients with IMAP in the VS and no VS group (Table 1). The mean SBP correlated with IMAP and was significantly increased in the VS (153 ± 11 mmHg vs. 137 ± 10 mmHg; p < 0.001) and NO VS group (154 ± 11 mmHg vs. 139 ± 13 mmHg; p < 0.001).

No correlation could be shown between the blood pressure levels during aSAH and the occurrence of radiological signs of pulmonary congestion (VS: p = 0.722/NO VS: p = 0.538, hereinafter), ACS (p > 0.99/p = 0.380) and impaired kidney function (p > 0.99/p = 0.172, see Fig. 2 and Table S2 in the supplementary materials).

## Discussion

This study aimed to elucidate the impact of different blood pressure values during the acute phase of aSAH on the outcome. We were able to show that IMAP correlates with poor functional outcome and occurrence of DCI associated infarction. Noteworthy is that in this study MAP ≥ 95 mmHg was not linked to an increase in cardiac complications and impairment of the kidney function.

The influence of increased blood pressure and respectively MAP on the outcome following aSAH has been discussed extensively^5,6,16^. Over the decades, induced hypertension was a component of the Triple H therapy and routinely utilized to target cerebral vasospasm^5^. Although this concept was increasingly abandoned since the 2000’s, hypertension remains the cornerstone element of conservative vasospasm management^3,4^, being at the same time controversially discussed^6^. In particular, the HIMALAIA trial revealed that extremely induced hypertension (up to MAP 130 mmHg or SBP 230 mmHg) does not translate into better outcome but more cardiac complications. Of note, the control group in this trial was kept over a MAP of 80 mmHg^16^.

In our study, IMAP beyond the therapeutic target independently correlated with unfavorable clinical and radiographic outcome of aSAH in individuals with cerebral vasospasm. At the same time, blood pressure values did not show impact on the study endpoints in patients that did not develop vasospasm.

An autonomous increase of the blood pressure levels during acute cerebrovascular events, including aSAH, was described before^17,18^. The time point of this blood pressure increase after aSAH was considered crucial for the final impact on the functional outcome and DCI risk^19^. Interestingly, especially early spontaneous increased blood pressure and persisting endogenous hypertension was associated with or showed a trend towards poor outcome and increased probability of DCI^19^. Furthermore, in case of an ischemic stroke, blood pressure will increase in the majority of patients and take up to 10 days to reach baseline levels^20–22^. The multiple progenitors leading to DCI associated infarction development after aSAH may cause a constant state of prolonged ischemia in different areas of the brain. Comparable to ischemic stroke, this seems to lead to a constant upregulation of the blood pressure. Moreover, aSAH leads to an impairment of cerebral autoregulation^23,24^. This might additionally contribute to poor outcome in patients that exceed the aimed MAP values.

The differences in the vasopressors dosage between SMAP and IMAP patients observed in the NO VS group might be related to the need for optimization of the CPP in the individuals with IMAP, since there was no vasospasm-triggered autonomous upregulation of the blood pressure in these patients. In contrast, IMAP pattern was not driven by higher dosage of vasopressors in aSAH individuals with cerebral vasospasm, as MAP increase over the therapeutic aim might be, at least partially, related to autoregulatory mechanisms in this cohort group.

The erring link between autonomous upregulation and IMAP in the NO VS group and the missing positive impact on DCI associated infarction and outcome, questions induced hypertension in the treatment of aSAH in patients without clinical vasospasm.

A higher blood pressure has been connected with increased cardiac and kidney-related complications, especially if caused iatrogenic in the acute phase of aSAH^16^. As mentioned before, an increase in cardiac complications was the main reason for early termination of the HIMALAIA trial^16^. However, in our cohort no correlation between cardiac and nephrological complications and IMAP could be shown. It might be due to the fact that the MAP values aimed in our cohort were not driven as high as in other studies on intention and that both study cohorts might not be equal regarding preexisting kidney and heart function. In summary, our results are in line with the previous studies and show that a moderate MAP management without utilization of extreme hypertension is a useful measure against increased risk of systemic cardiac, pulmonary and kidney-related complications.

### Limitations

This is a retrospective study that includes the common drawbacks of this study design. Even if based on a prospectively enrolling electronic database, the completeness and accuracy of the available dataset was lower as compared to studies with a complete prospective design. For two variables (GFR and pulmonary congestion) a relevant portion of the data was missing. Furthermore, due to the retrospective setting of the study without a standardized study protocol, we were unable to analyze the possible association between the MAP values at the specific timepoints and the subsequent clinical events. Accordingly, we limited the analyses to the mean values over a predefined time interval of two weeks that might better reflect the autoregulatory reaction of patients during aSAH. Of note, the analyses based on the maximum, minimum and delta values of the blood pressure did not show any significant results. Finally, there is a risk of bias regarding the treatment intention and outcome confounding which was addressed by utilizing subgroup analyses with further correction for relevant confounders in the multivariable analysis.

## Conclusion

Sustained increase of MAP beyond the therapeutic targets in patients with aSAH and cerebral vasospasm independently correlates with the risk of DCI associated infarction and poor functional outcome. Therefore, the presence of MAP exceeding the aimed values without additional iatrogenic intervention on the blood pressure might reflect the autoregulatory mechanisms against the impending cerebral ischemia in patients with cerebral vasospasm. aSAH patients with such vasospasm-triggered blood pressure upregulation might profit from intense diagnostic and preventive therapeutic interventions against cerebral vasospasm.

## Supplementary Information

Supplementary Information.

## Data Availability

The data that support the findings of this study are available from the corresponding author upon reasonable request.
