# Supplementary material for: Blood pressure and outcome after aneurysmal subarachnoid hemorrhage
Source: Sci Rep. 2022 May 14;12:8006. doi: 10.1038/s41598-022-11903-4 (PMC9107458; doi:10.1038/s41598-022-11903-4)
Supplement: Supplementary file 1 — Supplementary Information. [file 41598_2022_11903_MOESM1_ESM.pdf]

Supplements

Supplements figure S1: Dot-Plot of distribution of 14-days-mean MAP between the NO VS and VS group.

Abbreviations: IMAP – increased mean arterial pressure, (NO) VS – (no) vasospasm

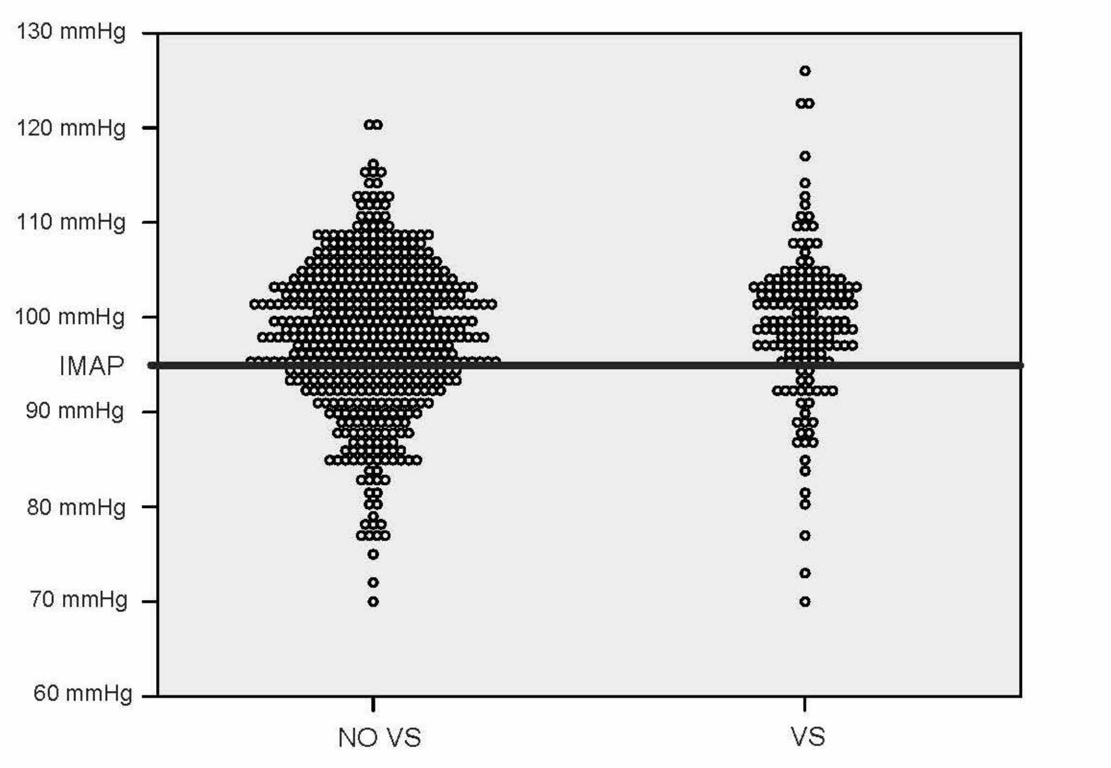

Supplements figure S2: Box-plots for total use of norepinephrine in mg during the first 14 days in IMAP and SMAP groups.

Abbreviations: (I/S)MAP – (increased/standard) mean arterial pressure, (NO) VS – (no) vasospasm

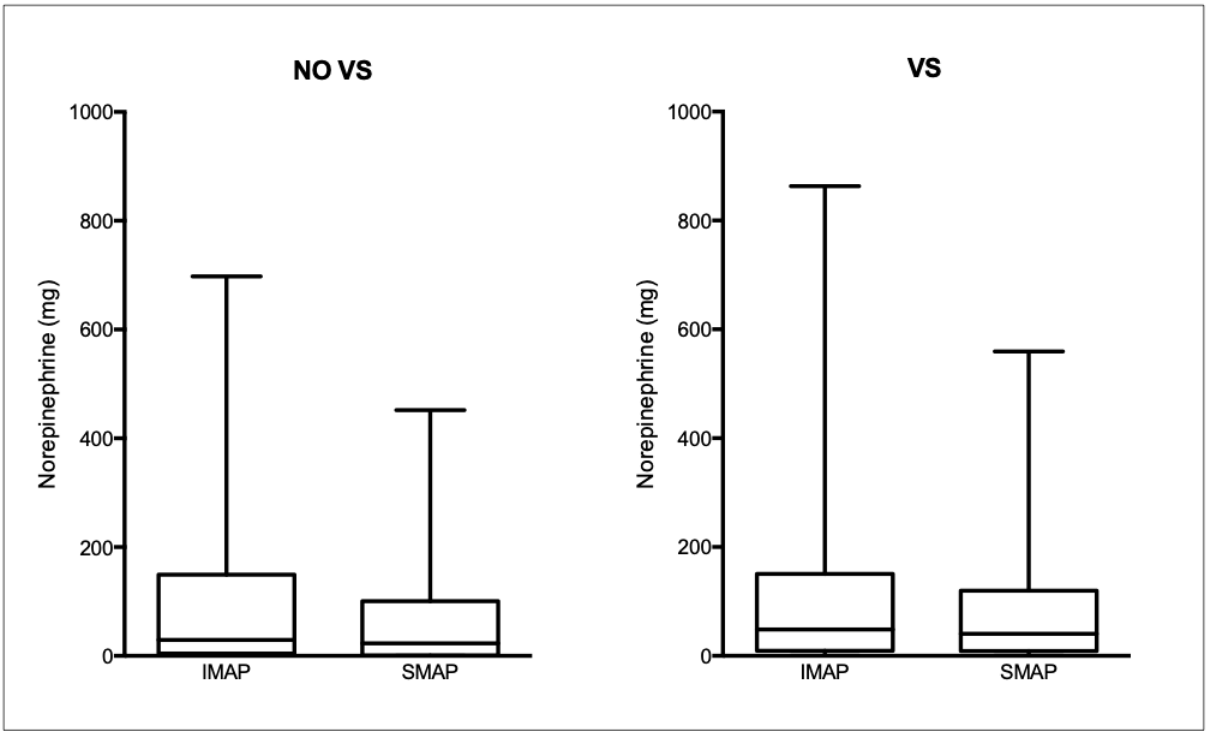

Parameter		***N/mean***	***%/SD***
Demographics	Age (years)	54	±14
	Sex female	464	67.2%
	Premorbid arterial hypertension	492	71.3%
aSAH characteritics	WFNS 4-5	310	44.9%
	Fisher 3-4 *	581	90.4%
Treatment	Clipping	285	41.3%
	ICP therapy †	327	47.5%
	Additional vasopressors ‡	74	10.8%
Complications	ACS	16	2.3%
	Pulmonal congestion (Shochat score >1) #	168	34.7%
	GFR Mean < 60mL/min/1.3m2 **	27	5.9%
Blood pressure	IMAP	474	68.7%
Outcome	Early infarct ***	209	30.3%
	DCI Infarct***	168	24.4%
	Poor outcome at 6 month follow-up****	328	48.0%

* Data missing for 47 patients
† Data missing for 2 patients
‡ Data missing for 7 patients
# Data missing for 205 patients
** Data missing for 238 patients
*** Data missing for 1 patients
**** Data missing for 6 patients

Supplements table S1: Overview of the cohort characteristics.

Parameter		***VS group***				
		SMAP/n =37	*IMAP/n=126*			
		%/mean +/- SD	%/mean +/- SD	p	OR	95% CI
Complications	ACS	0.0%	2.4%	>0.99	1.02	0.99-1.05
	Pulmonal congestion (Shochat score >1)	29.0%	32.4%	0.722	1.17	0.49-2.81
	GFR Mean < 60 mL/minute/1.73m^2	0.0%	2.0%	>0.99	1.02	0.99-1.05
Outcome	DCI Infarct	24.3%	46.8%	**0.015**	2.74	1.20-6.27
	Poor outcome at 6-month follow-up	37.8%	61.8%	**0.010**	2.66	1.25-5.67
		NO VS group				
		SMAP/n=179	IMAP/n=348			
		%	%	p	OR	95% CI
Complications	ACS	3.4%	2.0%	0.380	0.59	0.20-1.78
	Pulmonal congestion (Shochat score >1)	33.3%	36.8%	0.538	1.17	0.71-1.91
	GFR Mean < 60 mL/minute/1.73m^2	5.4%	9.6%	0.172	1.86	0.75-4.59
Outcome	DCI Infarct	12.3%	22.5%	**0.005**	2.07	1.24-3.45
	Poor outcome at 6-month follow-up	32.6%	52.0%	**<0.001**	2.24	1.54-3.27

Supplements table S2: Univariate analysis for primary and secondary endpoints separate for the vasospasm and no vasospasm group.
